# Prevalence of and Trends in the Co-Existence of Obesogenic Behaviors in Adolescents From 15 Countries

**DOI:** 10.3389/fped.2021.664828

**Published:** 2021-04-22

**Authors:** Hui Fan, Xingyu Zhang

**Affiliations:** ^1^Department of Preventive Medicine, North Sichuan Medical College, Nanchong, China; ^2^Applied Biostatistics Laboratory, University of Michigan School of Nursing, Ann Arbor, MI, United States

**Keywords:** prevalence, trend, adolescents, obesogenic behaviors, co-existence

## Abstract

**Background:** The global epidemic of pediatric obesity is well-known, but data on co-existence of obesogenic behaviors are limited. We aim to report the prevalence of and trends in the co-existence of obesogenic behaviors in adolescents from 15 countries.

**Methods:** This study was based on the Global School-based Student Health Survey 2003–2017 and included 121,963 adolescents aged 12–15 years from 15 countries where at least 2 cross-sectional surveys were conducted. We used sampling weights and calculated the country-level prevalence of and trends in the co-existence of obesogenic behaviors (low fruit and vegetable intake, anxiety-induced insomnia, no physical activity, and sedentary behavior) during survey years. Pooled prevalence and trend estimates were calculated with random-effects models.

**Results:** Pooled prevalence of exposure ≥ 1, ≥2, and ≥3 obesogenic behaviors was 88.2, 44.9, and 9.8% in the first survey and 88.4, 46.4, and 10.2% in the last survey, respectively. Plateauing, increasing, and decreasing trends in the co-existence of obesogenic behaviors were observed in different countries. Specifically, we identified a plateauing pooled trend in the exposure ≥ 1, ≥2, and ≥3 obesogenic behaviors [odds ratios (95% confidence intervals): 1.03 (0.93, 1.14), 1.05 (0.97, 1.13), and 1.06 (0.95, 1.18), respectively].

**Conclusion:** Trends in the prevalence of the co-existence of obesogenic behaviors varied significantly across different countries, but the prevalence remained high in most countries. These findings suggest the need for behavioral interventions to mitigate obesogenic behaviors in adolescents for overweight and obesity prevention.

## Introduction

Obesity in children and adolescents is a major risk factor for physical and psychosocial disorders throughout the lifespan (1–5). The prevalence of obesity in children and adolescents in recent years has been increasing in most countries, and has thus become a worldwide health concern (1, 2). During infectious disease pandemics, such as the COVID-19 pandemic, individuals with overweight/obesity who are infected are more likely to develop serious illnesses or die, compared with normal-weight individuals (6).

The onset and development of obesity is a complicated process affected by a variety of biological and behavioral factors (7, 8). Compared with biological factors (e.g., genetics), behavioral factors (i.e., obesogenic behaviors), which include poor dietary habits, anxiety-induced insomnia, no physical activity, and sedentary behavior, are easily preventable, identifiable, and modifiable. Obesogenic behaviors also play an important role in the occurrence of population-wide obesity epidemics (7, 8), given that they are common in children and adolescents, according to global cross-sectional studies (9, 10). Behavioral habits established during childhood and adolescence tend to persist into and consolidate in adulthood (11, 12). Therefore, establishing healthy behavioral habits in childhood and adolescence is crucial to curb the epidemic of obesity and prevent associated disorders.

Single obesogenic behavior has been clarified well in adolescents (9, 10, 13). However, to our best knowledge, data on prevalence of and trends in the co-existence of obesogenic behaviors in adolescents are limited. Such evidence is essential to develop and assess policies and programs to mitigate obesogenic behaviors. In this study, we aimed to assess the aforementioned issue based on the Global School-Based Student Health Survey (GSHS) 2003–2017.

## Materials and Methods

### Study Population

The GSHS is a series of multi-country, repeated cross-sectional, and school-based surveys (9, 10). It was launched by the World Health Organization and US Centers for Disease and Control and Prevention to investigate health behaviors among school-attending adolescents. It uses a standardized 2-stage, school-to-class probability sampling design in each participating country to obtain a nationally representative sample (9, 10). All students in the chosen class are invited to participate the survey. Data collection is conducted during a regular class period. Each country-specific GSHS during 2003–2017 was approved by the local government administration and an institutional review board or ethics committee. All participating students, parents, and/or school officials provided informed consent.

Publicly available data from GSHS can be found at https://www.cdc.gov/gshs and https://www.who.int/ncds/surveillance/gshs/factsheets/en/. From the available GSHS dataset, 34 countries that underwent repeated cross-sectional surveys were included in the present study. We excluded 1 country that lacked a nationally representative sample; 1 country with a different coding rule compared to other countries; 2 countries where the last surveys were not conducted after 2010; 11 countries that lacked data on fruit and vegetable intake, anxiety-induced insomnia, physical activity, sedentary behavior or weighted information; and 4 countries that lacked a sufficient sample size (≥1,000) in any of at least 2 survey waves. Consequently, 177,201 school-attending adolescents from 15 countries were included in this study. We then excluded 46,029 adolescents whose age was missing or were >15 or <12 years old, and 9,209 adolescents for whom data on sex, food insecurity, fruit and vegetable intake, anxiety-induced insomnia, physical activity, sedentary behavior or weighted information were lacking. Eventually, 121,963 adolescents aged 12–15 years were included in the final analysis.

### Measures and Definition

All students completed the survey questionnaire about obesogenic behaviors (14). Low vegetable and fruit intake was defined as consumption of fruit and vegetables <5 servings per day during the past 30 days (15). Participants, who were so worried about something that they could not sleep at night most of the time or always during the past 12 months, were considered to have anxiety-induced insomnia (9, 10). No physical activity was defined as no physical activity performance for at least 60 min on any day during the past 7 days (9, 10). Participants, who spend ≥3 h/day during a typical or usual day in sitting and watching television, playing computer games, talking with friends, or doing other sitting activities after excluding time at school or doing homework, were considered to have sedentary behaviors (9, 10).

The GSHS asked participants for their obesogenic behaviors (low fruit and vegetable intake, anxiety-induced insomnia, no physical activity, and sedentary behavior); a value of “1” or “0” was assigned to participants who had or did not have each type of obesogenic behavior, respectively. Consequently, the number of obesogenic behaviors for each participant ranged from 0 to 4, with each type of obesogenic behavior worth 1 point.

Data on sex, age, and food insecurity were also collected. Food insecurity was evaluated based on the question “How often did you go hungry because there was not enough food in your home during the past 30 days?” The response options were “never,” “rarely/sometimes,” and “most of the time/always (9, 10).”

### Statistical Analysis

Sampling weights were used to account for the complex sampling design (14). We calculated the country-specific prevalence of each type of obesogenic behavior in different survey years. We also evaluated the country-level prevalence of exposure ≥1, ≥2, and ≥3 obesogenic behaviors. We used the logistic regression analyses to assess trends in the prevalence of exposure ≥1 (reference: <1), ≥2 (reference: <2), and ≥3 (reference: <3) obesogenic behaviors across the survey years after adjustment for sex, age, and food insecurity. For example, to assess the trends in the prevalence of exposure ≥2 obesogenic behaviors, we assigned the outcome value of “1” or “0” to participants who had ≥2 or had <2 obesogenic behaviors, respectively. We computed the pooled prevalence in the first and last surveys by the corresponding country-specific prevalence using a random-effects model. To assess the pooled trend in the prevalence of exposure ≥1, ≥2, and ≥3 obesogenic behaviors across the survey years, we also used a random-effects model to calculate the pooled estimates stratified by WHO region (Americas, Eastern Mediterranean, Western Pacific, African, and Southeast Asia), World Bank income level (high income, upper-middle income, and lower-middle income), and number of surveys (2 and ≥3), based on the corresponding country-specific estimated odds ratio (OR) and 95% confidence interval (95% CI).

We used SAS version 9.4 (SAS Institute, Cary, NC, USA) and STATA version 11.0 (Stata Corporation, College Station, TX, USA) to perform the statistical analyses, and considered *P*-values of <0.05 as statistically significant.

## Results

This study included 121,963 adolescents aged 12–15 years from 15 countries. Table 1 summarizes the country-level survey characteristics during the survey years (2003–2017). The 15 participating countries were classified into 5 WHO regions (Southeast Asia: 4; Eastern Mediterranean: 4; Western Pacific: 3; Americas: 3; African: 1) and 3 World Bank income levels (high income: 4; upper-middle income: 7; lower-middle income: 4). Further, 4 and 11 countries had ≥3 and two survey waves, respectively. The GSHS in the 15 countries were conducted in different survey years from 2003 to 2017. The response rates for all country-level surveys were >85%. The country-level available sample sizes in the surveys varied, ranging from 1,024 in Seychelles in 2007 to 19,883 in Argentina in 2012.

**Table 1:** Survey characteristics in 15 countries.

**Country**	**Region**	**Income**	**Survey year**	**Response rate, %**	**Available sample size**	**Mean age, years**	**Boys, %**
Argentina	Americas	Upper middle income	2007	94.2	1,448	14.1	45.9
			2012	92.4	19,883	13.9	47.6
Fiji	Western Pacific	Upper middle income	2010	94.0	1,405	14.0	47.6
			2016	88.3	1,357	14.4	48.1
Guatemala	Americas	Upper middle income	2009	94.4	4,244	13.9	52.2
			2015	85.9	3,101	13.9	50.5
Indonesia	South-East Asia	Upper middle income	2007	96.9	2,928	13.8	49.2
			2015	95.2	8,384	13.5	49.1
Kuwait	Eastern Mediterranean	High income	2011	95.0	2,183	14.1	49.5
			2015	85.9	1,748	14.1	50.1
Lebanon	Eastern Mediterranean	Upper middle income	2011	93.0	1,843	13.7	46.5
			2017	89.6	3,000	13.6	46.0
Morocco	Eastern Mediterranean	Lower middle income	2006	92.3	1,834	14.0	52.3
			2010	91.7	2,206	13.7	53.3
			2016	88.4	3,513	13.6	50.5
Myanmar	South-East Asia	Lower middle income	2007	98.4	2,191	13.6	49.5
			2016	95.9	2,146	13.6	46.4
Philippines	Western Pacific	Lower middle income	2003	92.8	3,896	14.1	40.1
			2007	94.6	3,296	14.3	44.5
			2011	93.5	3,596	13.9	47.8
			2015	96.9	5,971	13.9	47.9
Seychelles	African	High income	2007	88.7	1,024	13.6	47.9
			2015	90.4	1,864	13.5	47.0
Sri Lanka	South-East Asia	Lower middle income	2008	96.8	2,423	13.7	49.6
			2016	95.8	2,159	14.0	48.8
Thailand	South-East Asia	Upper middle income	2008	97.3	2,603	13.6	47.7
			2015	92.9	3,839	13.7	48.4
Tonga	Western Pacific	Upper middle income	2010	94.8	1,844	14.1	50.3
			2017	94.1	1,945	13.6	51.3
Trinidad and Tobago	Americas	High income	2007	90.6	2,219	13.8	48.3
			2011	91.2	2,154	13.6	49.3
			2017	90.1	2,489	13.6	47.3
United Arab Emirates	Eastern Mediterranean	High income	2005	92.4	11,844	13.7	47.6
			2010	93.1	2,144	14.0	38.3
			2016	93.3	3,239	13.9	47.0

Table 2 shows the prevalence of each type of obesogenic behavior across the 15 countries in different survey years. The prevalence of low fruit and vegetable intake, anxiety-induced insomnia, no physical activity, and sedentary behavior varied widely, ranging from 52.6% in Seychelles in 2007 to 89.8% in Myanmar in 2016, from 1.8% in Myanmar in 2007 to 19.6% in Kuwait in 2011, from 14.1% in Argentina in 2012 to 45.4% in Philippines in 2007, and from 9.7% in Myanmar in 2007 to 63.3% in Kuwait in 2015, respectively. The respective pooled prevalence of these 4 obesogenic behaviors was 72.0, 10.5, 24.8, and 36.2% in the first survey and 73.1, 9.9, 25.7, and 37.2% in the last survey.

**Table 2:** Prevalence of each type of obesogenic behaviors across 15 countries in different survey year.

**Country**	**Survey year**	**Low fruit and vegetable intake, %**	**Anxiety-induced sleep problems, %**	**No physical activity, %**	**Sedentary behaviors, %**
Argentina	2007	85.8	10.9	22.9	49.5
	2012	82.4	8.4	14.1	50.3
Fiji	2010	60.6	14.5	26.4	27.5
	2016	63.4	11.2	28.5	28.1
Guatemala	2009	73.4	6.9	19.3	25.3
	2015	70.3	6.3	30.4	22.9
Indonesia	2007	75.2	7.5	14.7	33.8
	2015	75.3	4.3	34.2	24.4
Kuwait	2011	77.7	19.6	28.0	53.5
	2015	81.1	17.8	20.5	63.3
Lebanon	2011	71.7	10.5	16.3	47.2
	2017	75.7	12.0	22.1	40.2
Morocco	2006	62.9	13.4	16.7	30.1
	2010	52.9	14.1	22.2	25.9
	2016	64.3	14.0	23.7	26.3
Myanmar	2007	83.6	1.8	31.1	9.7
	2016	89.8	3.4	29.4	15.7
Philippines	2003	75.1	13.6	45.0	28.3
	2007	78.6	12.1	45.4	29.5
	2011	74.2	10.4	42.5	32.5
	2015	74.5	10.4	44.7	30.8
Seychelles	2007	52.6	11.9	27.1	51.1
	2015	59.3	10.5	30.8	49.4
Sri Lanka	2008	77.2	4.3	17.7	33.1
	2016	75.9	3.8	15.1	35.1
Thailand	2008	66.1	6.6	19.1	37.7
	2015	70.0	8.0	23.2	50.8
Tonga	2010	61.0	15.5	32.5	29.2
	2017	54.8	13.4	24.4	20.4
Trinidad and Tobago	2007	76.4	9.2	32.8	49.2
	2011	83.9	7.2	28.0	44.0
	2017	81.8	11.7	26.8	46.3
United Arab Emirates	2005	81.3	12.6	24.9	39.0
	2010	83.0	15.1	19.9	51.2
	2016	76.5	14.3	19.0	54.6
Pooled estimate	First survey	72.0	10.5	24.8	36.2
	Last survey	73.1	9.9	25.7	37.2

Table 3 presents the country-level prevalence of the co-existence of obesogenic behaviors and related trends across the survey years. The prevalence of exposure ≥1, ≥2, and ≥3 obesogenic behaviors differed substantially, ranging from 74.9% in Tonga in 2017 to 95.0% in Argentina in 2007, from 31.3% in Morocco in 2010 and in Tonga in 2017 to 66.0% in Kuwait in 2015, and from 2.7% in Myanmar in 2007 to 22.8% in Kuwait in 2011, respectively. Plateauing, increasing, and decreasing trends were observed in the prevalence of exposure ≥2 obesogenic behaviors in 10, 3, and 2 countries, respectively (*P*_s_ > 0.05 for 10 countries; *P*_s_ < 0.05 for 3 and 2 countries). Notably, the prevalence of the co-existence of ≥2 obesogenic behaviors in all of the 15 countries in the latest survey year remained high (>30%), regardless of the different country-level trends. Furthermore, plateauing, increasing, and decreasing trends were identified in the prevalence of ≥3 obesogenic behaviors in 8, 5, and 2 countries, respectively (*P*_s_ > 0.05 for 8 countries; *P*_s_ < 0.05 for 5 and 2 countries). In 4 of the 8 countries with a plateauing trend (Trinidad and Tobago, Kuwait, United Arab Emirates, and the Philippines) and 3 of the 5 countries with an increasing trend (Lebanon, Seychelles, and Thailand), the prevalence of ≥3 obesogenic behaviors in the latest survey year was >10%. Supplementary Tables 1 and 2 describe the country-level prevalence and trend among boys and girls, respectively. The difference in the trend among boys and girls can be noted in Indonesia, Kuwait, Lebanon, Seychelles, and United Arab Emirates.

**Table 3:** Prevalence of the co-existence of obesogenic behaviors and their trends in 15 countries across survey years.

**Country**	**Survey year**	**Number of obesogenic behaviors≥1**			**Number of obesogenic behaviors≥2**			**Number of obesogenic behaviors≥3**		
		**Prevalence, %**	OR (95%CI)^*^	***P***	**Prevalence, %**	OR (95%CI)^*^	***P***	**Prevalence, %**	OR (95%CI)^*^	***P***
Argentina			0.73 (0.49, 1.10)	0.134		0.77 (0.61, 0.96)	0.023		0.70 (0.60, 0.80)	<0.001
	2007	95.0			58.6			13.9		
	2012	93.1			51.8			9.7		
Fiji			0.95 (0.73, 1.24)	0.700		1.08 (0.90, 1.30)	0.396		1.05 (0.79, 1.40)	0.736
	2010	84.4			37.9			6.4		
	2016	83.7			40.0			7.0		
Guatemala			1.02 (0.89, 1.16)	0.823		1.18 (0.96, 1.45)	0.117		1.06 (0.72, 1.58)	0.754
	2009	84.7			34.4			5.5		
	2015	85.0			38.4			6.0		
Indonesia			1.09 (0.90, 1.32)	0.380		1.33 (1.08, 1.63)	0.007		1.09 (0.77, 1.55)	0.635
	2007	88.2			36.6			6.1		
	2015	88.5			43.1			6.3		
Kuwait			1.30 (0.77, 2.21)	0.310		1.27 (0.95, 1.71)	0.105		0.81 (0.63, 1.05)	0.103
	2011	93.2			60.5			22.8		
	2015	94.7			66.0			19.5		
Lebanon			1.03 (0.82, 1.29)	0.802		1.13 (0.97, 1.32)	0.120		1.38 (1.09, 1.74)	0.010
	2011	89.3			45.8			9.4		
	2017	89.2			47.8			12.0		
Morocco			1.14 (1.03, 1.26)	0.015		1.06 (0.99, 1.13)	0.101		1.31 (1.13, 1.50)	<0.001
	2006	81.5			35.5			5.7		
	2010	77.4			31.3			6.0		
	2016	83.5			36.0			8.3		
Myanmar			1.71 (1.18, 2.49)	0.006		1.19 (0.97, 1.46)	0.096		2.38 (1.54, 3.67)	<0.001
	2007	88.7			34.7			2.7		
	2016	92.9			38.8			6.1		
Philippines			1.01 (0.93, 1.10)	0.825		0.98 (0.93, 1.03)	0.339		0.99 (0.92, 1.06)	0.737
	2003	92.3			56.0			12.8		
	2007	93.7			57.6			13.1		
	2011	91.6			55.1			11.8		
	2015	92.7			54.5			12.3		
Seychelles			1.18 (1.02, 1.37)	0.028		1.14 (1.00, 1.29)	0.045		1.18 (1.01, 1.39)	0.042
	2007	85.3			45.2			11.4		
	2015	87.2			48.7			13.0		
Sri Lanka			0.82 (0.65, 1.04)	0.098		0.96 (0.80, 1.15)	0.638		0.97 (0.72, 1.30)	0.817
	2008	89.0			37.6			5.6		
	2016	87.2			37.0			5.5		
Thailand			1.70 (1.28, 2.26)	0.001		1.56 (1.22, 2.00)	<0.001		1.51 (1.13, 2.04)	0.007
	2008	81.9			39.0			8.2		
	2015	88.7			50.3			12.1		
Tonga			0.55 (0.44, 0.68)	<0.001		0.63 (0.54, 0.74)	<0.001		0.68 (0.54, 0.86)	0.001
	2010	84.7			43.4			9.5		
	2017	74.9			31.3			6.4		
Trinidad and Tobago			1.10 (0.95, 1.27)	0.219		0.98 (0.89, 1.07)	0.665		0.98 (0.87, 1.10)	0.688
	2007	92.2			57.9			15.7		
	2011	94.5			54.4			13.1		
	2017	93.0			56.7			15.0		
United Arab Emirates			0.90 (0.79, 1.02)	0.097		1.06 (0.99, 1.13)	0.114		1.05 (0.97, 1.15)	0.231
	2005	91.8			51.3			13.3		
	2010	93.8			57.9			15.8		
	2016	91.1			56.0			15.6		

* *Adjusting for sex, age, food insecurity; CI, confidence interval; OR, odds ratio*.

Table 4 reports the pooled estimates of the prevalence of and trends in the co-existence of obesogenic behaviors from the 15 countries. The pooled prevalence of ≥1, ≥2, and ≥3 obesogenic behaviors was 88.2, 44.9, and 9.8% in the first survey and 88.4, 46.4, and 10.2% in the last survey, respectively. A plateauing pooled trend was observed in the prevalence of ≥1, ≥2, and ≥3 obesogenic behaviors [OR (95% CI): 1.03 (0.93, 1.14), 1.05 (0.97, 1.13), and 1.06 (0.95, 1.18), respectively].

**Table 4:** Pooled estimate about prevalence and trend in the co-existence of obesogenic behaviors.

	**Number of obesogenic behaviors ≥1**			**Number of obesogenic behaviors ≥2**			**Number of obesogenic behaviors ≥3**		
	**Prevalence on first survey, %**	**Prevalence on last survey, %**	**OR (95%CI)**	**Prevalence on first survey, %**	**Prevalence on last survey, %**	**OR (95%CI)**	**Prevalence on first survey, %**	**Prevalence on last survey, %**	**OR (95%CI)**
All countries (*n* = 15)	88.2	88.4	1.03 (0.93, 1.14)	44.9	46.4	1.05 (0.97, 1.13)	9.8	10.2	1.06 (0.95, 1.18)
**WHO region**									
Americas (*n* = 3)	90.6	90.4	1.02 (0.88, 1.17)	50.2	49.0	0.97 (0.80, 1.17)	11.6	10.2	0.88 (0.67, 1.15)
Eastern Mediterranean (*n* = 4)	89.0	89.7	1.04 (0.89, 1.21)	48.2	51.3	1.07 (1.02, 1.12)	12.6	13.8	1.12 (0.93, 1.35)
Western Pacific (*n* = 3)	87.2	83.8	0.81 (0.55, 1.19)	45.8	41.9	0.88 (0.66, 1.16)	9.5	8.5	0.90 (0.70, 1.14)
African (*n* = 1)	85.3	87.2	1.18 (1.02, 1.37)	45.2	48.7	1.14 (1.00, 1.29)	11.4	13.0	1.18 (1.01, 1.39)
South-East Asia (*n* = 4)	87.1	89.3	1.25 (0.89, 1.74)	36.9	42.2	1.23 (1.01, 1.50)	5.6	7.5	1.37 (0.96, 1.95)
**Income**									
High income (*n* = 4)	90.6	91.5	1.07 (0.91, 1.24)	53.5	56.6	1.06 (0.99, 1.15)	15.3	15.6	1.02 (0.92, 1.14)
Upper middle income (*n* = 7)	87.0	86.2	0.96 (0.76, 1.21)	42.0	43.2	1.05 (0.83, 1.33)	8.4	8.4	1.02 (0.78, 1.32)
Lower middle income (*n* = 4)	87.9	89.2	1.08 (0.91, 1.27)	41.0	41.6	1.03 (0.96, 1.10)	6.6	8.1	1.24 (0.96, 1.61)
**Number of surveys**									
2 (*n* = 11)	87.7	87.8	1.03 (0.87, 1.23)	42.9	44.7	1.08 (0.93, 1.25)	9.1	9.3	1.07 (0.88, 1.31)
≥3 (*n* = 4)	91.8	90.3	1.03 (0.94, 1.14)	50.1	50.8	1.02 (0.97, 1.07)	11.8	12.7	1.06 (0.96, 1.18)

*CI, confidence interval; OR, odds ratio*.

## Discussion

This study indicated that the prevalence of exposure ≥ 2 and ≥3 obesogenic behaviors varied widely across the 15 countries and continued to be at high levels in most countries. We also founded the plateauing, increasing, and decreasing trends in the prevalence of exposure ≥ 2 and ≥3 obesogenic behaviors within different countries.

The effects of obesogenic behaviors on adiposity are well-known. For example, low consumption of fruits and vegetables is known to promote pediatric obesity due to the displacement of fiber- and micronutrient-rich fruits and vegetables with energy-dense foods (16). A short sleep duration is believed to play an important role in the onset and development of obesity through changes in endocrinal, neurological, and behavioral mechanisms (17). Low levels of physical activity, which reduce energy expenditure and promote appetite, are also implicated in obesity (18). Sedentary behavior is reported to contribute to obesity via snacking, decreasing energy expenditure, and increasing abdominal fat (19).

Previous cross-sectional studies based on GSHS dataset and publications from other data sources have reported a high prevalence of single obesogenic behaviors (e.g., low fruit and vegetable intake, insufficient physical activity, and sedentary behavior), which is consistent with our results (9, 10, 19–22). However, our findings further reveal that the prevalence of the co-existence of ≥2 and ≥3 obesogenic behaviors varied widely across the included countries during the survey years (2003–2017), but remained high in most countries regardless of survey waves. Further, compared with previous studies that evaluated trends in single obesogenic behaviors (13, 23, 24), the added advantage of this study is that it included only those countries that had at least 2 GSHS waves (15 countries in total) and could thus demonstrate dynamic country-level changes in the co-occurrence of obesogenic behaviors. Our study also revealed plateauing, increasing, and decreasing trends in the prevalence of the co-existence of ≥2 and ≥3 obesogenic behaviors across different countries. These results are partly consistent with those of previous studies that observed trends in the prevalence of insufficient physical activity, low fruit and vegetable intake, and sedentary behavior (13, 23, 24).

Our finding of the wide variation in the co-occurrence of ≥2 and with the ≥3 obesogenic behaviors and the corresponding trends across countries might be attributable to country-level variations in environmental, economic, social, policy-related, and other factors (7, 8, 13, 25–29). For example, the epidemics of obesogenic behaviors are partly driven by changes in the food environment (e.g., availability of unhealthy processed foods), economic progress (e.g., industrialization, urbanization, marketization, and globalization), and social transformations (e.g., technological advances such as the informatization and introduction of activity-saving technologies) (26–29). With regard to policy-related factors, some countries that recognize the urgent need to tackle childhood obesity have implemented policies to mitigate obesogenic behaviors, with noticeable effects (1, 2, 13, 26, 27, 30). This explains the decreasing trend in the co-existence of obesogenic behaviors we observed in some countries.

Multilevel factors, such as individual, family, community, and societal factors, contribute to the onset and development of childhood obesity (31). Notably, family, community, and societal factors tend to contribute to pediatric obesity through individual-level obesogenic behaviors (31). Genetic predisposition, another individual-level factor, is partly responsible for obesity in childhood, but is believed to play a minor role relative to behavioral factors (18). Taken together, these findings suggest that the prevention of and interventions to mitigate obesogenic behaviors are crucial in curbing the epidemic of childhood obesity. Our findings have important implications for mitigating obesogenic behaviors and related-obesity in adolescents. First, the results suggest that country-level prevention and intervention policies for obesogenic behaviors should be established and implemented in countries where relevant policies are scarce. Second, in the remaining countries where the prevalence has increased or remained high, it is necessary to reconsider the effectiveness of existing policies. Third, obesogenic behavior prevention and intervention policies should be comprehensive, such that they target poor dietary habits, sleep disorders, low/no physical activity, and sedentary behavior (7, 8, 26). Finally, sex differences in the trend in the prevalence of the co-existence of obesogenic behaviors in some countries should be noted for policy makers. The reason for these results needs to be clarified.

The main strengths of our study are the nationally representative samples, rigorous and standardized data collection, the practical nature of this research and its global scale. However, some limitations need to be acknowledged. First, this study did not include countries from Europe or low-income countries, included only 1 country from the African region, and excluded 11 countries without complete data and 4 countries without the sufficient sample size (≥1,000) during any of at least 2 survey waves, which limits the generalizability of our findings. Second, the survey years, sample sizes, and survey numbers differed between the participating countries, which could have affected our results. Third, our study included only school-attending adolescents aged 12–15 years. A future study on trends in obesogenic behaviors should focus on younger children or adolescents who do not attend school. Fourth, there is no data about environmental factors that affect obesogenic behavior in the present study. Finally, the data collected were self-reported, which may have introduced some bias.

In conclusion, the results of this study indicate that the prevalence of the co-existence of obesogenic behaviors remained high in most of the included countries during 2003–2017, even though the corresponding trends varied widely across the countries. This finding suggests that most countries need to implement interventions to mitigate obesogenic behaviors in adolescents and prevent overweight and obesity.

## Data Availability Statement

The raw data supporting the conclusions of this article will be made available by the authors, without undue reservation.

## Ethics Statement

The studies involving human participants were reviewed and approved by the local government administration and an institutional review board or ethics committee. Written informed consent to participate in this study was provided by the participants' legal guardian/next of kin.

## Author Contributions

HF conceptualized and designed the study, carried out the initial analyses, drafted the initial manuscript, and reviewed and revised the manuscript. XZ critically reviewed and revised the manuscript. All authors contributed to the article and approved the submitted version.

## Conflict of Interest

The authors declare that the research was conducted in the absence of any commercial or financial relationships that could be construed as a potential conflict of interest.

## Abbreviations

GSHS: global school-based student health surveys
WHO: world health organization.
