# Supplementary material for: Prevalence of and Trends in the Co-Existence of Obesogenic Behaviors in Adolescents From 15 Countries
Source: Front Pediatr. 2021 Apr 22;9:664828. doi: 10.3389/fped.2021.664828 (PMC8100235; doi:10.3389/fped.2021.664828)
Supplement: Supplementary file 1 [file Table_1.DOCX]

| Table S1. Prevalence of the [co-existence](javascript:;) of obesogenic behaviors and their trends in 15 countries among boys | | | | | | | | | | | | |
| --- | --- | --- | --- | --- | --- | --- | --- | --- | --- | --- | --- | --- |
| Country | Survey year | Number of obesogenic behaviors≥1 | | |  | Number of obesogenic behaviors≥2 | | |  | Number of obesogenic behaviors≥3 | | |
|  |  | Prevalence, % | OR (95%CI)* | *P* |  | Prevalence, % | OR (95%CI)* | *P* |  | Prevalence, % | OR (95%CI)* | *P* |
| Argentina |  |  | 0.65 (0.38, 1.14) | 0.130 |  |  | 0.95 (0.73, 1.22) | 0.678 |  |  | 0.72 (0.54, 0.95) | 0.022 |
|  | 2007 | 94.9 |  |  |  | 50.5 |  |  |  | 8.9 |  |  |
|  | 2012 | 92.2 |  |  |  | 49.0 |  |  |  | 6.3 |  |  |
| Fiji |  |  | 0.90 (0.60, 1.34) | 0.585 |  |  | 1.09 (0.84, 1.41) | 0.510 |  |  | 0.96 (0.65, 1.44) | 0.854 |
|  | 2010 | 85.2 |  |  |  | 39.1 |  |  |  | 7.0 |  |  |
|  | 2016 | 84.0 |  |  |  | 40.6 |  |  |  | 7.0 |  |  |
| Guatemala |  |  | 1.26 (0.95, 1.66) | 0.106 |  |  | 1.27 (0.99, 1.64) | 0.063 |  |  | 0.74 (0.38, 1.44) | 0.363 |
|  | 2009 | 84.2 |  |  |  | 32.6 |  |  |  | 4.4 |  |  |
|  | 2015 | 87.0 |  |  |  | 38.5 |  |  |  | 3.4 |  |  |
| Indonesia |  |  | 1.04 (0.80, 1.35) | 0.788 |  |  | 1.41 (1.10, 1.80) | 0.007 |  |  | 1.42 (0.92, 2.17) | 0.111 |
|  | 2007 | 88.4 |  |  |  | 35.0 |  |  |  | 4.7 |  |  |
|  | 2015 | 88.2 |  |  |  | 42.9 |  |  |  | 6.3 |  |  |
| Kuwait |  |  | 1.74 (0.87, 3.46) | 0.113 |  |  | 1.56 (1.07, 2.29) | 0.023 |  |  | 0.99 (0.65, 1.51) | 0.961 |
|  | 2011 | 91.3 |  |  |  | 53.7 |  |  |  | 16.3 |  |  |
|  | 2015 | 94.7 |  |  |  | 63.4 |  |  |  | 16.0 |  |  |
| Lebanon |  |  | 0.95 (0.73, 1.24) | 0.687 |  |  | 1.11 (0.90, 1.38) | 0.327 |  |  | 1.44 (0.95, 2.19) | 0.088 |
|  | 2011 | 87.5 |  |  |  | 41.7 |  |  |  | 6.2 |  |  |
|  | 2017 | 86.5 |  |  |  | 43.4 |  |  |  | 8.5 |  |  |
| Morocco |  |  | 1.10 (0.96, 1.25) | 0.159 |  |  | 1.10 (1.00, 1.21) | 0.059 |  |  | 1.43 (1.19, 1.71) | <0.001 |
|  | 2006 | 81.7 |  |  |  | 32.2 |  |  |  | 4.8 |  |  |
|  | 2010 | 78.3 |  |  |  | 29.5 |  |  |  | 4.7 |  |  |
|  | 2016 | 83.2 |  |  |  | 34.7 |  |  |  | 7.9 |  |  |
| Myanmar |  |  | 1.79 (1.17, 2.74) | 0.008 |  |  | 1.27 (1.01, 1.61) | 0.045 |  |  | 1.81 (1.12, 2.93) | 0.017 |
|  | 2007 | 88.9 |  |  |  | 32.3 |  |  |  | 2.9 |  |  |
|  | 2016 | 93.4 |  |  |  | 37.7 |  |  |  | 5.2 |  |  |
| Philippines |  |  | 1.07 (0.96, 1.19) | 0.199 |  |  | 1.02 (0.96, 1.09) | 0.555 |  |  | 1.02 (0.93, 1.11) | 0.673 |
|  | 2003 | 91.3 |  |  |  | 53.2 |  |  |  | 11.2 |  |  |
|  | 2007 | 92.3 |  |  |  | 55.3 |  |  |  | 10.8 |  |  |
|  | 2011 | 91.8 |  |  |  | 54.4 |  |  |  | 10.8 |  |  |
|  | 2015 | 92.7 |  |  |  | 54.7 |  |  |  | 11.4 |  |  |
| Seychelles |  |  | 1.04 (0.84, 1.29) | 0.729 |  |  | 0.98 (0.83, 1.17) | 0.845 |  |  | 1.23 (0.94, 1.60) | 0.137 |
|  | 2007 | 85.8 |  |  |  | 43.6 |  |  |  | 8.6 |  |  |
|  | 2015 | 86.0 |  |  |  | 43.6 |  |  |  | 10.1 |  |  |
| Sri Lanka |  |  | 0.78 (0.57, 1.07) | 0.118 |  |  | 1.09 (0.89, 1.34) | 0.401 |  |  | 0.95 (0.59, 1.53) | 0.843 |
|  | 2008 | 90.5 |  |  |  | 36.0 |  |  |  | 5.8 |  |  |
|  | 2016 | 88.0 |  |  |  | 38.4 |  |  |  | 5.6 |  |  |
| Thailand |  |  | 2.05 (1.41, 2.99) | <0.001 |  |  | 1.60 (1.21, 2.11) | 0.001 |  |  | 1.51 (1.07, 2.13) | 0.020 |
|  | 2008 | 81.1 |  |  |  | 37.9 |  |  |  | 7.5 |  |  |
|  | 2015 | 89.8 |  |  |  | 49.5 |  |  |  | 10.8 |  |  |
| Tonga |  |  | 0.56 (0.41, 0.77) | <0.001 |  |  | 0.58 (0.46, 0.73) | <0.001 |  |  | 0.66 (0.47, 0.94) | 0.020 |
|  | 2010 | 84.4 |  |  |  | 45.5 |  |  |  | 9.5 |  |  |
|  | 2017 | 75.4 |  |  |  | 32.2 |  |  |  | 6.6 |  |  |
| Trinidad and Tobago |  |  | 1.08 (0.87, 1.34) | 0.496 |  |  | 1.01 (0.88, 1.17) | 0.863 |  |  | 0.98 (0.84, 1.16) | 0.842 |
|  | 2007 | 91.3 |  |  |  | 51.8 |  |  |  | 11.3 |  |  |
|  | 2011 | 93.1 |  |  |  | 50.4 |  |  |  | 10.0 |  |  |
|  | 2017 | 92.1 |  |  |  | 52.4 |  |  |  | 11.3 |  |  |
| United Arab Emirates |  |  | 0.86 (0.75, 0.99) | 0.034 |  |  | 1.03 (0.95, 1.12) | 0.433 |  |  | 1.01 (0.91, 1.12) | 0.883 |
|  | 2005 | 90.5 |  |  |  | 46.4 |  |  |  | 9.5 |  |  |
|  | 2010 | 91.3 |  |  |  | 50.0 |  |  |  | 10.9 |  |  |
|  | 2016 | 88.4 |  |  |  | 49.4 |  |  |  | 10.1 |  |  |
| *Adjusting for age, food insecurity; CI, confidence interval; OR, odds ratio. | | | | |  |  |  |  |  |  |  |  |

| Table S2. Prevalence of the [co-existence](javascript:;) of obesogenic behaviors and their trends in 15 countries among girls | | | | | | | | | | | | |
| --- | --- | --- | --- | --- | --- | --- | --- | --- | --- | --- | --- | --- |
| Country | Survey year | Number of obesogenic behaviors≥1 | | |  | Number of obesogenic behaviors≥2 | | |  | Number of obesogenic behaviors≥3 | | |
|  |  | Prevalence, % | OR (95%CI)* | *P* |  | Prevalence, % | OR (95%CI)* | *P* |  | Prevalence, % | OR (95%CI)* | *P* |
| Argentina |  |  | 0.82 (0.53, 1.26) | 0.366 |  |  | 0.63 (0.47, 0.85) | 0.002 |  |  | 0.68 (0.55, 0.85) | <0.001 |
|  | 2007 | 95.0 |  |  |  | 65.5 |  |  |  | 18.1 |  |  |
|  | 2012 | 94.0 |  |  |  | 54.4 |  |  |  | 12.7 |  |  |
| Fiji |  |  | 0.99 (0.62, 1.58) | 0.977 |  |  | 1.07 (0.86, 1.34) | 0.517 |  |  | 1.13 (0.74, 1.73) | 0.569 |
|  | 2010 | 83.6 |  |  |  | 36.8 |  |  |  | 5.9 |  |  |
|  | 2016 | 83.4 |  |  |  | 39.5 |  |  |  | 7.1 |  |  |
| Guatemala |  |  | 0.83 (0.63, 1.09) | 0.174 |  |  | 1.09 (0.83, 1.43) | 0.554 |  |  | 1.31 (0.90, 1.91) | 0.156 |
|  | 2009 | 85.2 |  |  |  | 36.3 |  |  |  | 6.6 |  |  |
|  | 2015 | 82.9 |  |  |  | 38.3 |  |  |  | 8.6 |  |  |
| Indonesia |  |  | 1.15 (0.86, 1.52) | 0.347 |  |  | 1.26 (0.99, 1.59) | 0.057 |  |  | 0.88 (0.59, 1.32) | 0.540 |
|  | 2007 | 88.0 |  |  |  | 38.2 |  |  |  | 7.4 |  |  |
|  | 2015 | 88.8 |  |  |  | 43.3 |  |  |  | 6.2 |  |  |
| Kuwait |  |  | 0.92 (0.53, 1.59) | 0.751 |  |  | 1.02 (0.77, 1.37) | 0.867 |  |  | 0.70 (0.54, 0.91) | 0.010 |
|  | 2011 | 95.0 |  |  |  | 67.2 |  |  |  | 29.2 |  |  |
|  | 2015 | 94.7 |  |  |  | 68.6 |  |  |  | 23.1 |  |  |
| Lebanon |  |  | 1.14 (0.85, 1.52) | 0.387 |  |  | 1.15 (0.94, 1.40) | 0.182 |  |  | 1.35 (1.04, 1.77) | 0.028 |
|  | 2011 | 90.9 |  |  |  | 49.3 |  |  |  | 12.1 |  |  |
|  | 2017 | 91.6 |  |  |  | 51.6 |  |  |  | 15.0 |  |  |
| Morocco |  |  | 1.18 (1.04, 1.34) | 0.011 |  |  | 1.02 (0.91, 1.14) | 0.717 |  |  | 1.21 (0.99, 1.49) | 0.069 |
|  | 2006 | 81.2 |  |  |  | 39.1 |  |  |  | 6.8 |  |  |
|  | 2010 | 76.5 |  |  |  | 33.3 |  |  |  | 7.5 |  |  |
|  | 2016 | 83.8 |  |  |  | 37.3 |  |  |  | 8.6 |  |  |
| Myanmar |  |  | 1.64 (1.01, 2.66) | 0.044 |  |  | 1.11 (0.86, 1.44) | 0.411 |  |  | 3.00 (1.62, 5.55) | <0.001 |
|  | 2007 | 88.5 |  |  |  | 37.2 |  |  |  | 2.5 |  |  |
|  | 2016 | 92.6 |  |  |  | 39.8 |  |  |  | 6.9 |  |  |
| Philippines |  |  | 0.96 (0.86, 1.08) | 0.491 |  |  | 0.95 (0.89, 1.00) | 0.063 |  |  | 0.97 (0.89, 1.06) | 0.516 |
|  | 2003 | 92.9 |  |  |  | 57.8 |  |  |  | 13.9 |  |  |
|  | 2007 | 94.8 |  |  |  | 59.5 |  |  |  | 14.9 |  |  |
|  | 2011 | 91.5 |  |  |  | 55.6 |  |  |  | 12.7 |  |  |
|  | 2015 | 92.7 |  |  |  | 54.4 |  |  |  | 13.1 |  |  |
| Seychelles |  |  | 1.32 (1.10, 1.59) | 0.004 |  |  | 1.30 (1.11, 1.52) | 0.002 |  |  | 1.15 (0.96, 1.39) | 0.128 |
|  | 2007 | 84.9 |  |  |  | 46.7 |  |  |  | 13.9 |  |  |
|  | 2015 | 88.2 |  |  |  | 53.1 |  |  |  | 15.5 |  |  |
| Sri Lanka |  |  | 0.85 (0.59, 1.22) | 0.375 |  |  | 0.85 (0.65, 1.11) | 0.216 |  |  | 0.98 (0.68, 1.42) | 0.928 |
|  | 2008 | 87.5 |  |  |  | 39.1 |  |  |  | 5.3 |  |  |
|  | 2016 | 86.4 |  |  |  | 35.7 |  |  |  | 5.4 |  |  |
| Thailand |  |  | 1.43 (1.05, 1.96) | 0.026 |  |  | 1.53 (1.13, 2.08) | 0.008 |  |  | 1.52 (1.05, 2.21) | 0.029 |
|  | 2008 | 82.7 |  |  |  | 40.0 |  |  |  | 9.0 |  |  |
|  | 2015 | 87.5 |  |  |  | 51.0 |  |  |  | 13.3 |  |  |
| Tonga |  |  | 0.53 (0.41, 0.69) | <0.001 |  |  | 0.69 (0.56, 0.85) | <0.001 |  |  | 0.69 (0.50, 0.96) | 0.030 |
|  | 2010 | 85.1 |  |  |  | 41.4 |  |  |  | 9.5 |  |  |
|  | 2017 | 74.3 |  |  |  | 30.4 |  |  |  | 6.1 |  |  |
| Trinidad and Tobago |  |  | 1.12 (0.85, 1.48) | 0.407 |  |  | 0.95 (0.84, 1.08) | 0.448 |  |  | 0.98 (0.83, 1.15) | 0.784 |
|  | 2007 | 93.0 |  |  |  | 63.5 |  |  |  | 19.8 |  |  |
|  | 2011 | 95.8 |  |  |  | 58.4 |  |  |  | 16.1 |  |  |
|  | 2017 | 93.9 |  |  |  | 60.5 |  |  |  | 18.3 |  |  |
| United Arab Emirates |  |  | 0.96 (0.80, 1.15) | 0.621 |  |  | 1.08 (0.98, 1.19) | 0.130 |  |  | 1.08 (0.97, 1.21) | 0.182 |
|  | 2005 | 93.1 |  |  |  | 55.8 |  |  |  | 16.8 |  |  |
|  | 2010 | 95.3 |  |  |  | 62.8 |  |  |  | 18.9 |  |  |
|  | 2016 | 93.5 |  |  |  | 61.9 |  |  |  | 20.4 |  |  |
| *Adjusting for age, food insecurity; CI, confidence interval; OR, odds ratio. | | | | |  |  |  |  |  |  |  |  |
